# Cryopreservation of Endothelial Cells in Various Cryoprotective Agents and Media – Vitrification versus Slow Freezing Methods

**DOI:** 10.1371/journal.pone.0149660

**Published:** 2016-02-18

**Authors:** Achim von Bomhard, Alexander Elsässer, Lucas Maximilian Ritschl, Silke Schwarz, Nicole Rotter

**Affiliations:** 1 Technical University of Munich, Department of Maxillofacial Surgery, Langerstr. 3, 81675 Munich, Germany; 2 University Medical Center Ulm, Department of Otorhinolaryngology, Frauensteige 12, 89075 Ulm, Germany; University of California at Berkeley, UNITED STATES

## Abstract

Vitrification of endothelial cells (MHECT-5) has not previously been compared with controlled slow freezing methods under standardized conditions. To identify the best cryopreservation technique, we evaluated vitrification and standardized controlled-rate -1°C/minute cell freezing in a -80°C freezer and tested four cryoprotective agents (CPA), namely dimethyl sulfoxide (DMSO), ethylene glycol (EG), propylene glycol (PG), and glycerol (GLY), and two media, namely Dulbecco's modified Eagle medium Ham’s F-12 (DMEM)and K^+^-modified TiProtec (K^+^TiP), which is a high-potassium-containing medium. Numbers of viable cells in proliferation were evaluated by the CellTiter 96® A_Queous_ One Solution Cell Proliferation Assay (Promega Corporation, Mannheim, Germany). To detect the exact frozen cell number per cryo vial, DNA content was measured by using Hoechst 33258 dye prior to analysis. Thus, results could be evaluated unconstrained by absolute cell number. Thawed cells were cultured in 25 cm^2^ cell culture flasks to confluence and examined daily by phase contrast imaging. With regard to cell recovery immediately after thawing, DMSO was the most suitable CPA combined with K^+^TiP in vitrification (99 ±0.5%) and with DMEM in slow freezing (92 ±1.6%). The most viable cells in proliferation after three days of culture were obtained in cells vitrificated by using GLY with K^+^TiP (308 ±34%) and PG with DMEM in slow freezing (280 ±27%).

## Introduction

The endothelium is the monolayer of endothelial cells lining the lumen of all blood vessels. Disorders in the endothelium predispose the vessel wall to vasoconstriction, leukocyte adherence, platelet activation, mitogenesis, pro-oxidation, thrombosis, impaired coagulation, vascular inflammation, and atherosclerosis [1]. To be able to study all these vascular disorders in vitro, the cryopreservation, storage, and shipment of endothelial cells is extremely important. Relevant cell culture models are also crucial to the study of the pathobiology of the lung microvascular endothelium and for an understanding of lung metastasis [2] and disorders such as acute respiratory distress syndrome [3, 4]. Large numbers of quality-controlled cells are required for in vitro research and could, potentially, be used for cell therapy in clinical application [5].

Cryopreservation with controlled slow cooling rate was first described by Polge, Smith and Parkes in 1949 using the protective properties of glycerol (GLY) [6]. Dimethyl sulfoxide (DMSO) was proposed as a cryoprotectant in 1960 by Lovelock & Bishop and was rapidly shown to have far more widespread applicability than glycerol, particularly for the preservation of cells in tissue culture [7]. The literature of the 1950s and 1960s is dominated by reports of efforts to achieve or improve the cryopreservation of a variety of cell types through empirical variations of freezing rates, thawing rates, cryoprotectant concentrations, and associated solutes [8]. Nevertheless, a recovery of viability of more than 80–90% is still rarely achieved.

Slow cooling avoids intracellular ice buildup, which can cause the rupture of the cell membrane at temperatures between 0°C and −70°C [9]. However, the formation of extracellular ice can still result in the dehydration of the cells. To prevent this, an ideal cooling rate should be chosen, and a cryoprotective agent (CPA) added [10]. CPAs are divided into intracellular agents, which penetrate inside the cells and prevent ice crystal formation and membrane rupture, and extracellular compounds that do not penetrate the cell membrane and act by reducing the hyperosmotic effect present during the freezing procedure [10]. DMSO, ethylene glycol (EG), propylene glycol (PG), and GLY are intracellular CPAs. Among the extracellular compounds are sucrose, trehalose, dextrose, and polyvinylpyrrolidone [11]. DMSO is the most commonly used CPA. It provides a high rate of postfreezing cell survival but presents chemical cytotoxicity at room temperature and can damage the cells osmotically [12]. This cytotoxicity forces the experimenter to work rapidly. Moreover, different workers require different amounts of time to thaw their cells completely and to wash them free of CPA. Therefore, less cytotoxic CPAs are needed for the standardization of such procedures.

Over the last few years, another fast freezing method has become increasingly popular: vitrification. This promising and easy process avoids damage to the cells caused by their dehydration during slow freezing. To prevent intracellular ice buildup, a higher concentration of CPA is necessary, but its cytotoxic effect can be reduced by immediate freezing in liquid nitrogen (LN_2_). Vitrification has not previously been compared with controlled slow freezing methods under standardized conditions for endothelial cells. Many reports have shown that the toxicity to cells not only depends on the chemical properties of CPAs, but also on the cell types to be frozen. The ways in which the various CPAs and their combinations affect a given cell type cannot be predicted, and hence, the selection of cryoprotectants for each type of cell is still largely dependent on an empirical and experimental approach [13].

To identify the best cryopreservation technique for endothelial cells, four CPAs, namely DMSO, EG, PG, and GLY, and two different types of medium were tested in two different cryopreservation protocols (slow freezing vs. vitrification).

## Materials and Methods

All reagents, cell culture media, and supplements were purchased from Sigma Aldrich (Munich, Germany), Invitrogen (Darmstadt, Germany), and Biochrom (Berlin, Germany), unless indicated otherwise. Cell culture material was obtained from Greiner Bio One (Frickenhausen, Germany).

### Derivation and culture of endothelial cells

Murine heart endothelial cells (MHEC5-T, Leibniz Institute DSMZ—German Collection of Microorganisms and Cell Cultures, Braunschweig, Germany) were used in this study. Cell culture was performed as recommended by DSMZ at 37°C and 5% CO_2_. Cells were seeded at 0.1 x10^5^ cells/cm^2^ in monolayer culture.

### Cryopreservation solution (CPS), vitrification solution (VS), and cell culture media

DMEM Ham’s F-12 + 2mM L-glutamine (Gln+) and K^+^TiP were tested as carrier solutions. K^+^TiP was prepared by supplementing 500mL TiProtec (Dr. Franz Köhler Chemie GmbH, Bensheim, Germany) with 0.871g K_2_SO_4_ and 3.87g 56.3% w/w potassium lactate and was diluted with 50mL distilled water. Table 1 shows the final composition of K^+^TiP.

**Table 1:** Composition of K^+^TiP.

Cl-	94	mM
H2PO4-	1	mM
α-ketoglutarate	2	mM
aspartate	5	mM
Na+	21	mM
K+	131	mM
Mg2+	7	mM
Ca2+	0.02	mM
N-acetylcysteine	27	mM
glycine	9	mM
alanine	5	mM
tryptophan	2	mM
saccharose	27	mM
glucose	9	mM
Desferal	0.075	mM
LK 614	0.015	mM
osmolarity	338	mosm/l
pH	6.93	

K^+^TiP was prepared by supplementing 500 mL TiProtec (Dr. Franz Köhler Chemie GmbH, Bensheim, Germany) with 0.871g K_2_SO_4_ and 3.87g 56.3% w/w potassium lactate and was diluted with 50mL distilled water prior to use. Table 1 shows the final composition of K^+^TiP.

Prior to freezing or vitrification, the cryopreservation solution (CPS) and vitrification solution (VS) were prepared containing foetal bovine serum (FBS), sucrose (in VS), and one of the CPAs.

CPS was used for the controlled-rate -1°C/minute freezing. It contained one of the two tested carrier solutions, 40% FBS, and 3M of one of the following CPAs: DMSO, EG, PG, or GLY. For freezing, CPS was added 1:1 to cells in precooled carrier solution; therefore, the final concentration of FBS was 20% and of each CPA was 1.5M.

VS was used for vitrification. It contained one of the two tested carrier solutions, 40% FBS, 1M sucrose, and 6M of one of the following CPAs: DMSO, EG, PG, or GLY. For vitrification, the VS was added 1:1 to cells in precooled carrier solution; therefore, the final concentration of FBS was 20%, that of sucrose was 0.5M, and that of each CPAs was 3M. DMSO, GLY, and EG are reported to lead reliably to vitrification with tolerable toxicity at a concentration of 3M [14]. PG was also set to 3M in order to compare its efficacy.

The cell culture media and warming solution was DMEM Ham’s F-12 Gln+ supplemented with 10% FBS. Table 2 shows the features of the four cryoprotective agents used.

**Table 2:** Cryoprotective agents.

	dimethyl sulfoxide (DMSO)	ethylene glycol (EG)	glycerol (GLY)	propylene glycol (PG)	
**molar mass (M)**	78.13	62.07	92.09	76.09	g/mol
**density**	1.19	1.11	1.26	1.04	g/cm3
**purity**	0.999	0.999	0.999	0.999	%
**molarity**	15.21579419	17.86515225	13.66858508	13.65435668	mol/L

Table 2 shows the features of the four cryoprotective agents used.

### Cryopreservation and thawing

MHEC5-Ts were frozen at a final concentration of 10^6^ cells/mL by two different methods. At 80% confluence in Monolayer culture, the cells were trypsinized and counted, and 1 x10^6^ cells were transferred in 500μL precooled 4°C DMEM Ham’s F-12 Gln+ or 500μL precooled 4°C K^+^TiP into cryo vials. In the controlled-rate -1°C/minute freezing protocol, 500μL CPS was added, the cap was immediately tightened, and the vials were placed in a CoolCell (BioCision, Mill Valley, USA) in a −80°C freezer, left over night, and transferred into liquid nitrogen (LN_2_) the next day. Alternatively, for vitrification, 500μL VS was added, the cap was tightened, and vials were directly plunged into LN_2_. The whole process was performed within less than 15 seconds per vial. The samples were stored for 3 and 6 months.

Thawing was carried out quickly in a water bath at 37°C. To accelerate the warming process and to dilute the concentration of CPA, 1mL 37°C warming solution was added. Immediately after thawing, all samples were washed with 40mL DMEM Ham’s F-12 Gln+ to remove residual CPA, pelleted by centrifugation at 200x g for 5min, and resuspended in 2mL culture media. After repetitive gentle vortexing, 1mL of this cell suspension was used to determine the exact cell number by using Hoechst 33258. Aliquots of 500μL were used to determine cell viability at day 0, 1, 2, and 3. The residual 500μL of each sample was plated in a 25cm^2^ cell culture flask.

Ice formation during vitrification and thawing was evaluated by visual inspection for the presence of a milky appearance as described by Sheffen et al. [15].

### Determination of cell viability, number, recovery, and cell doubling time

The CellTiter 96® A_Queous_ One Solution Cell Proliferation Assay (COS, Promega Corporation, Mannheim, Germany) was used to determine viable cells in proliferation at one hour after thawing and at day one, two, and three. COS reagent contains a novel tetrazolium compound [3-(4,5-dimethylthiazol-2-yl)-5-(3-carboxymethoxyphenyl)-2-(4-sulfophenyl)-2H-tetrazolium, inner salt; MTS] and an electron-coupling reagent (phenazine ethosulfate; PES). The MTS tetrazolium compound (Owen’s reagent) is bioreduced by cells into a colored formazan product that is soluble in tissue culture medium. This conversion is presumed to be accomplished by NADPH or NADH produced by dehydrogenase enzymes in metabolically active cells [16]. Aliquots of 20μL of the COS reagent were pipetted directly into transparent 96-well cell culture plates containing endothelial cells in 100μL culture media and incubated for 1.5 hours. The absorbance was then recorded at 490nm with a 96-well plate reader (Infinite 200 PRO series, Tecan Group Ltd., Männedorf, Switzerland).

To detect the exact cell number per cryo vial, DNA content was measured according to Kim et al. by using Hoechst 33258 dye [17]. Cells were digested by using 0.5mg/mL proteinase K (AppliChem, Darmstadt, Germany) in 30mM Tris-HCl pH 8.0 at 56°C in a thermomixer comfort (Eppendorf, Hamburg, Germany) at 1000rpm. The digest was centrifuged at 2500x g for 5min. Aliquots of 100μL of each supernatant were diluted 1:5 in TNE-buffer (10mM Tris, 1mM Na_2_EDTA, 0.1mM NaCl, pH 7.4), and 500μL Hoechst 33258 dye 0.1pg/mL in TNE-buffer was added. After 20min, fluorescence was measured in triplicate in a black 96-well plate with a 96-well plate reader (Infinite® 200 PRO series, Tecan Group Ltd., Männedorf, Switzerland). The excitation wavelength was 356nm with a bandpass of 9nm, and the emission wavelength was 465nm with a bandpass of 20nm. Blanks contained 0.5mg/mL proteinase K in 30mM Tris-HCl pH 8.0 diluted 1:5 with TNE. For the determination of cell number, a calibration curve with MHEC5-Ts was performed. Samples were analyzed on day 0 (each n = 6).

Recovered MHEC5-Ts are able to attach to the culture flask bottom after thawing. Therefore, the cell recovery rate (cells attached to the flask / cells seeded x100%) was calculated by counting all dead cells in the supernatant after 24 hours by using a Neubauer chamber (Carl Roth, Karlsruhe, Germany) and trypan blue.

MHEC-5Ts doubling time could be analyzed by comparing COS–assay values immediately after thawing and on day 3 by using a standard curve.

The 25cm^2^ cell culture flasks were analyzed microscopically and photographed in six specific areas under phase contrast on a microscope (Axio Observer A1 inverted stand, Carl Zeiss GmbH, Jena, Germany) every day until confluence was reached. Cell numbers on day one till four were counted in these photographs with adobe illustrator CS5 (Adobe Systems, Delaware, USA).

### Statistical analysis

The two-tailed Mann-Whitney test was performed by using GraphPad Prism (version 6.0b for Mac OS X, GraphPad Software, La Jolla, USA). Values were considered significant when *p < 0*.05 (*), medium significant when *p < 0*.01 (**), highly siginificant when *p < 0*.001 (***), and of the highest siginificance when *p < 0*.0001 (****).

## Results

### Standardized controlled-rate -1°C/minute cell freezing in a -80°C freezer

**Fig 1:**
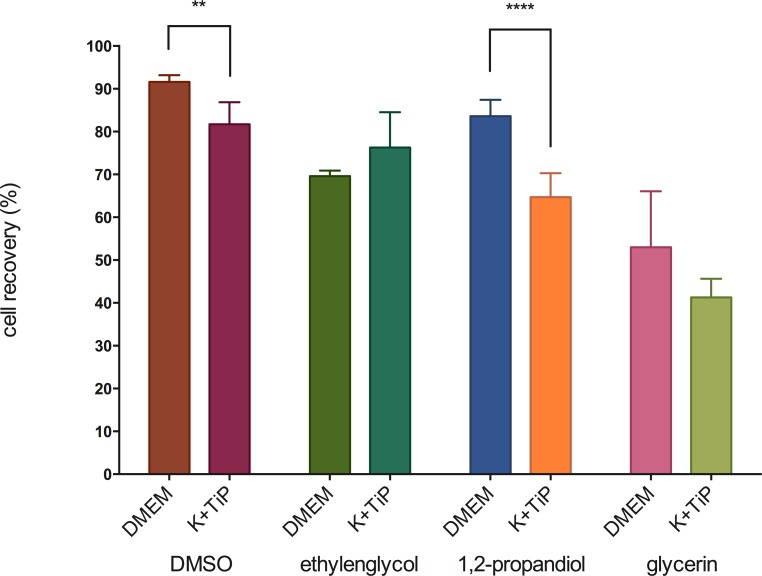
Cell recovery by using slow freezing methods. Figure 1 illustrates cell recovery of slowly frozen MHEC-5Ts immediately after thawing. DMEM = DMEM Ham’s F-12 Gln+, 20% FBS, and 1.5M of one of the CPAs; K^+^TiP = K^+^TiP. 0.5M sucrose, 20% FBS, and 1.5M of one of the CPAs; ** = medium significant (p < 0.01); **** = highest siginificance (p < 0.0001).

On thawing, cell recovery of MHEC-5Ts that had been frozen utilizing DMEM Ham’s F-12 Gln+ as the cryopreservation medium was 91.6 ±1.6% with DMSO, 83.6 ±3.8% with PG, 69.6 ±1.3% with EG, and 53.0 ±13.1% with GLY as the CPA. On thawing, cell recovery of MHEC-5Ts that had been frozen utilizing K^+^TiP as a cryopreservation medium was 81.7 ±5.2% with DMSO, 76.3 ±8.3% with EG, 64.7 ±5.6% with PG, and 41.3 ±4.4% with GLY as the CPA. Fig 1 illustrates these findings.

The numbers of viable cells in proliferation were determined colorimetrically. The highest absorbance at 1.5 hours after thawing relative to the cell count following the use of DMEM Ham’s F-12 Gln+ as the cryopreservation medium was 0.62 ±0.06 x10^-7^ absorbance units (au) (59 ±5.9% relative to unfrozen cells) with DMSO, and then 0.52 ±0.03 x10^-7^au (48.8 ±3%) with PG, 0.44 ±0.02 x10^-7^ au (41.6 ±2.1%) with EG, and 0.18 ±0.04 x10^-7^au (17.2 ±3.9%) with GLY as the CPA. The highest absorbance after thawing following the use of K^+^TiP as the cryopreservation medium was 0.65 ±0.01 x10^-7^au (62.4 ±1.2%) with DMSO, and then 0.54 ±0.03 x10^-7^au (50.8 ±2.7%) with PG, 0.43 ±0.09 x10^-7^au (41 ±8.4%) with EG, and 0.14 ±0.03 x10^-7^au (13.6 ±2.6%) with GLY as the CPA.

At 1 day after thawing, the highest absorbance utilizing DMEM Ham’s F-12 Gln+ as the cryopreservation medium was 1.03 ±0.14 x10^-7^au (97.8 ±12.8%) with DMSO, and then 0.83 ±0.11 x10^-7^au (78.2 ±10.1%) with PG, 0.7 ±0.02 x10^-7^au (66.3 ±1.8%) with EG, and 0.14 ±0.02 x10^-7^au (13.1 ±2.3%) with GLY as the CPA. The highest absorbance after thawing utilizing K^+^TiP as cryopreservation medium was 0.77 ±0.09 x10^-7^au (74.5 ±8.4%) with DMSO, and then 0.61 ±0.03 x10^-7^au (58.2 ±2.5%) with PG, 0.61 ±0.15 x10^-7^au (57.6 ±14.3%) with EG, and 0.15 ±0.03 x10^-7^au (13.7 ±2.6%) with GLY as the CPA.

Two days after thawing, the highest absorbance utilizing DMEM Ham’s F-12 Gln+ as the cryopreservation medium was 2.21 ±0.21 x10^-7^au (209.5 ±20.3%) with DMSO, and then 2.03 ±0.24 x10^-7^au (192 ±22.5%) with PG, 1.68 ±0.03 x10^-7^au (159.3 ±2.5%) with EG, and 0.17 ±0.03 x10^-7^au (15.7 ±2.9%) with GLY as the CPA. The highest absorbance after thawing utilizing K^+^TiP as cryopreservation medium was 1.94 ±0.18 x10^-7^au (183.3 ±16.8%) with DMSO, and then 1.56 ±0.07 x10^-7^au (147.9 ±6.7%) with PG, 1.5 ±0.37 x10^-7^au (141.8 ±35.4%) with EG, and 0.2 ±0.04 x10^-7^au (19.1 ±3.5%) with GLY as the CPA.

**Fig 2:**
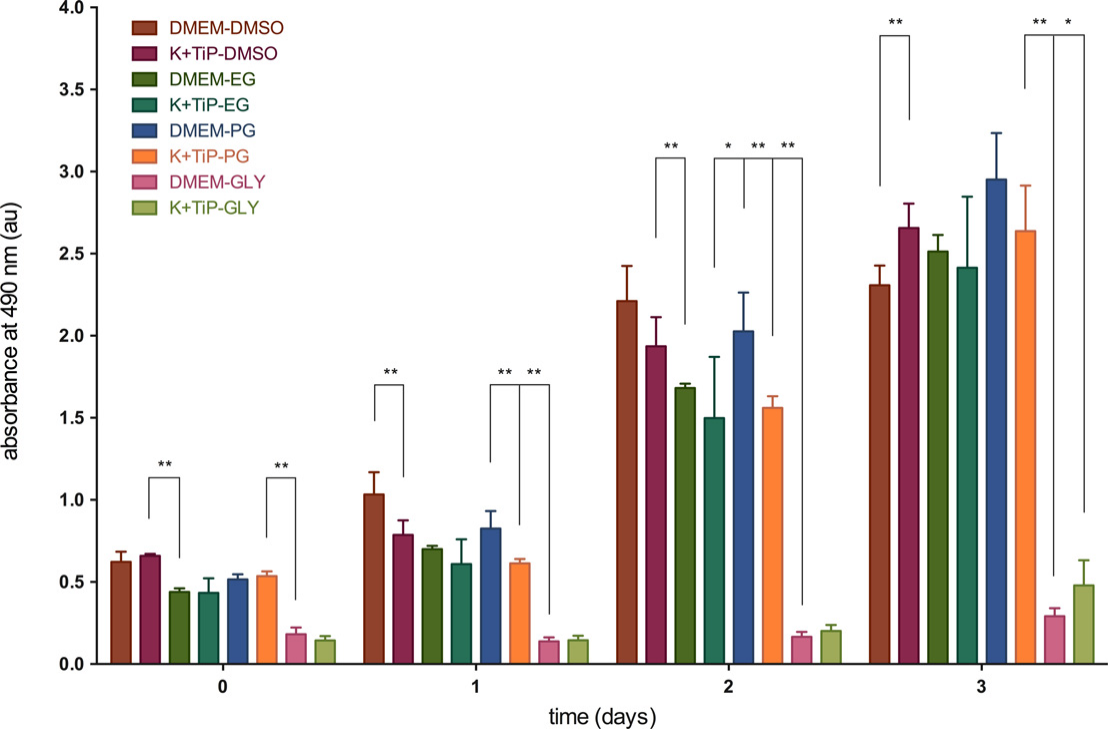
Viable cells in proliferation after use of slow freezing methods. The CellTiter 96® A_Queous_ One Solution Cell Proliferation Assay (COS, Promega Corporation, Mannheim, Germany) was used to determine viable cells in proliferation at one hour after thawing and at day one, two, and three. Figure 2 illustrates the absorption of viable cells in proliferation after the use of slow freezing methods. DMEM = DMEM Ham’s F-12 Gln+, 20% FBS, and 1.5M of one of the CPAs; K^+^TiP = K^+^TiP. 0.5M sucrose, 20% FBS, and 1.5M of one of the CPAs; DMSO = dimethyl sulfoxide; EG = ethylene glycol; PG = propylene glycol; GLY = glycerin; * = significant (p < 0.05); ** = medium significant (p < 0.01); au = absorbance units.

Three days after thawing, the highest absorbance utilizing DMEM Ham’s F-12 Gln+ as the cryopreservation medium was 2.95 ±0.28 x10^-7^au (279.6 ±26.9%) with PG, and then 2.5 ±0.1 x10^-7^au (238.1 ±9.5%) with EG, 2.31 ±0.12 x10^-7^au (218.6 ±11.4%) with DMSO, and 0.29 ±0.05 x10^-7^au (27.7 ±4.5%) with GLY as the CPA. The highest absorbance after thawing utilizing K^+^TiP as the cryopreservation medium was 2.66 ±0.15 x10^-7^au (251.7 ±14.1%) with DMSO, and then 2.64 ±0.28 x10^-7^au (249.8 ±26.3%) with PG, 2.41 ±0.43 x10^-7^au (228.6 ±41%) with EG, and 0.48 ±0.15 x10^-7^au (45.4 ±14.5%) with GLY as the CPA. Fig 2 illustrates these findings.

**Fig 3:**
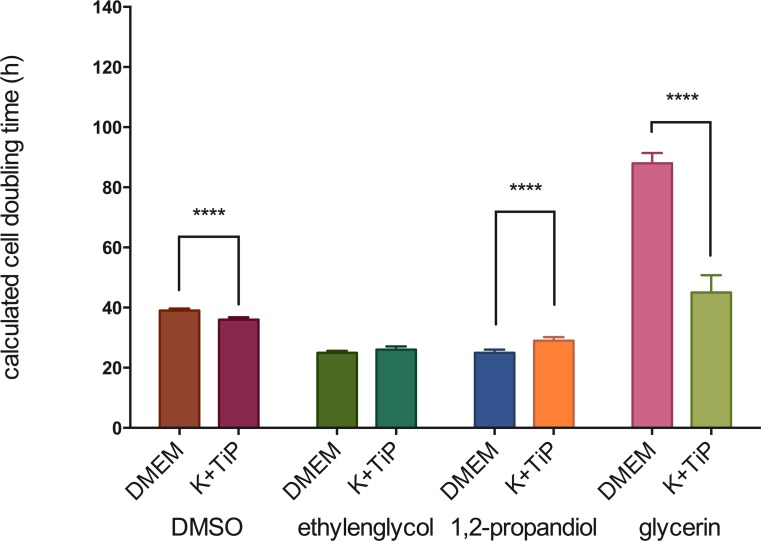
Cell doubling-time after the use of slow freezing methods. Figure 3 illustrates MHEC-5T doubling-time after the use of slow freezing methods. DMEM = DMEM Ham’s F-12 Gln+, 20% FBS, and 1.5M of one of the CPAs; K^+^TiP = K^+^TiP. 0.5M sucrose, 20% FBS, and 1.5M of one of the CPAs; **** = highest siginificance (p < 0.0001).

The fastest MHEC-5T doubling-time following the use of DMEM Ham’s F-12 Gln+ as the cryopreservation medium was 25 ±0.6h with EG, and then 25 ±1h with PG, 39 ±0.7h with DMSO, and 88 ±3.4h with GLY as the CPA. Doubling-time utilizing K^+^TiP as the cryopreservation media was 26 ±1.1h with EG, 29 ±1.2h with PG, 36 ±0.8h with DMSO, and 45 ±5.8h with GLY as the CPA. Fig 3 illustrates these findings.

All frozen cells could be cultured until confluence in the 25cm^2^ cell culture flasks after thawing. Confluence was reached on day 4 by cells previously frozen utilizing DMEM Ham’s F-12 Gln+ as the cryopreservation medium and DMSO as the CPA and utilizing K^+^TiP as the cryopreservation medium and DMSO or EG as the CPA. On day 5, confluence was reached by cells previously frozen utilizing DMEM Ham’s F-12 Gln+ as the cryopreservation medium and EG or PG as the CPA and utilizing K^+^TiP as the cryopreservation medium and PG as the CPA. On day 6, confluence was reached by cells previously frozen by utilizing K^+^TiP as the cryopreservation medium and GLY as the CPA. On day 8, confluence was reached by cells previously frozen by utilizing DMEM Ham’s F-12 Gln+ as the cryopreservation medium and GLY as the CPA.

### Vitrification

**Fig 4:**
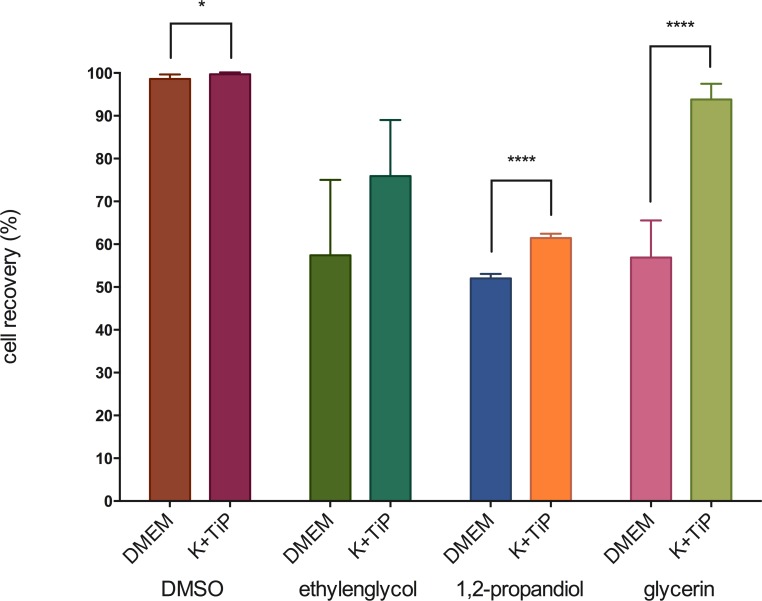
Cell recovery by using vitrification. Figure 4 illustrates cell recovery of vitrified MHEC-5Ts immediately after thawing. DMEM = DMEM Ham’s F-12 Gln+, 20% FBS, and 3M of one of the CPAs; K^+^TiP = K^+^TiP. 0.5M sucrose, 20% FBS, and 3M of one of the CPAs; * = significant (p < 0.05); **** = highest siginificance (p < 0.0001).

After thawing, cell recovery of MHEC-5Ts frozen by utilizing DMEM Ham’s F-12 Gln+ as the cryopreservation medium was 98.6 ±1.1% with DMSO, 57.4 ± 17.6% with EG, 56.9 ± 8.6% with GLY, and 52.0 ± 1.0% with PG as the CPA. After thawing, cell recovery of MHEC-5Ts frozen by utilizing K^+^TiP as the cryopreservation medium was 99.7 ±0.5% with DMSO, 93.8 ± 3.6% with GLY, 75.9 ± 13.1% with EG, and 61.4 ± 1.0% with PG as the CPA. Fig 4 illustrates these findings.

The numbers of viable cells in proliferation were determined colorimetrically. The highest absorbance relative to cell count 1.5 hours after the thawing of cells frozen by utilizing DMEM Ham’s F-12 Gln+ as cryopreservation media was 0.85 ±0.05 x10^-7^au (80.7 ±4.9% relative to unfrozen cells) with DMSO, and then 0.16 ±0.04 x10^-7^au (14.7 ±3.3%) with GLY, 0.11 ±0.01 x10^-7^au (10 ±0.6%) with PG, and 0.1 ±0.01 x10^-7^au (9.2 ±1.2%) with EG as the CPA. The highest absorbance after thawing following the use of K^+^TiP as the cryopreservation medium was 0.98 ±0.06 x10^-7^au (92.6 ±5.4%) with DMSO, and then 0.41 ±0.05 x10^-7^au (38.7 ±4.8%) with GLY, 0.19 ±0.05 x10^-7^au (17.5 ±5%) with PG, and 0.08 ±0.01 x10^-7^au (7.2 ±1%) with EG as the CPA.

At 1 day after thawing, the highest absorbance of cells previously frozen by utilizing DMEM Ham’s F-12 Gln+ as the cryopreservation media was 1.8 ±0.09 x10^-7^au (170.8 ±8.7% relative to unfrozen cells) with DMSO, and then 0.19 ±0.05 x10^-7^au (17.9 ±5.1%) with GLY, 0.09 ±0.01 x10^-7^au (8.6 ±0.8%) with EG, and 0.08 ±0.01 x10^-7^au (8 ±1%) with PG as the CPA. The highest absorbance after thawing cells previously frozen by utilizing K^+^TiP as the cryopreservation medium was 1.93 ±0.03 x10^-7^au (182.7 ±3.1%) with DMSO, and then 0.74 ±0.13 x10^-7^au (70.4 ±12.6%) with GLY, 0.18 ±0.06 x10^-7^au (17 ±6.1%) with PG, and 0.06 ±0.01 x10^-7^au (6 ±0.8%) with EG as the CPA.

Two days after thawing, the highest absorbance of cells frozen by utilizing DMEM Ham’s F-12 Gln+ as the cryopreservation medium was 2.9 ±0.2 x10^-7^au (274.3 ±18.6% relative to unfrozen cells) with DMSO, and then 0.38 ±0.14 x10^-7^au (35.7 ±13.6%) with GLY, 0.14 ±0.01 x10^-7^au (12.9 ±0.5%) with PG, and 0.13 ±0.03 x10^-7^au (12.3 ±2.5%) with EG as the CPA. The highest absorbance after the thawing of cells frozen by utilizing K^+^TiP as the cryopreservation medium was 2.83 ±0.16 x10^-7^au (268.2 ±15%) with DMSO, and then 2.28 ±0.26 x10^-7^au (216.1 ±25%) with GLY, 0.24 ±0.05 x10^-7^au (22.5 ±4.9%) with PG, and 0.1 ±0.03 x10^-7^au (9.2 ±2.8%) with EG as the CPA.

**Fig 5:**
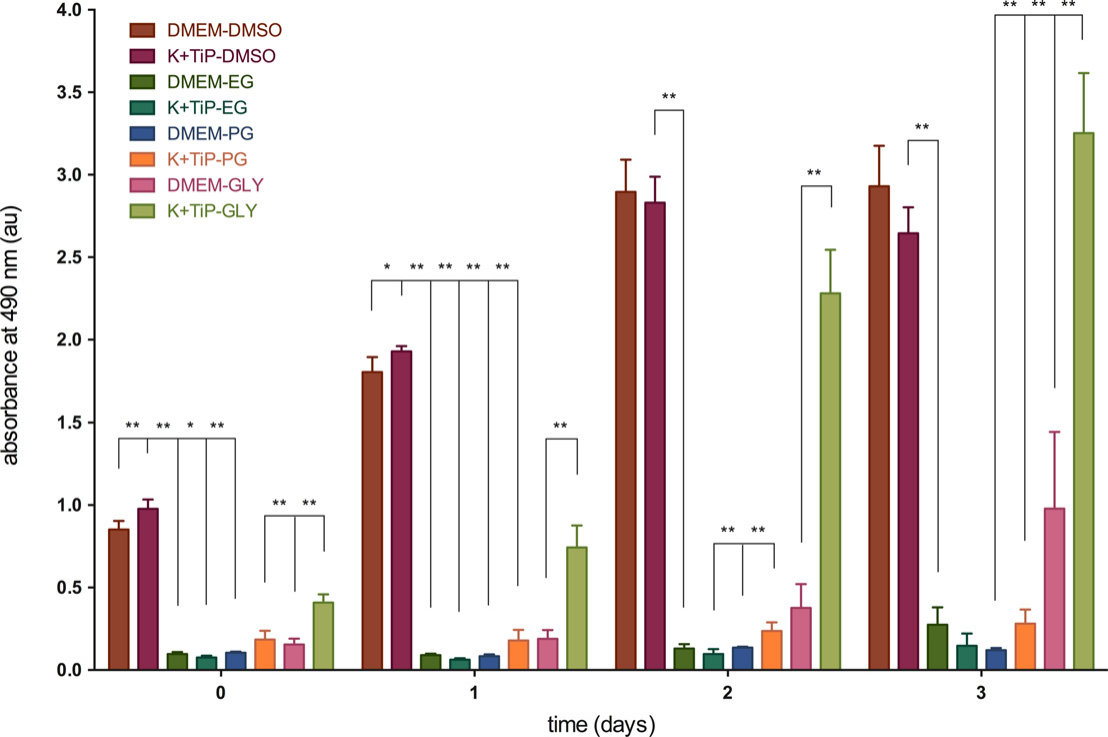
Viable cells in proliferation after vitrification. The CellTiter 96® A_Queous_ One Solution Cell Proliferation Assay (COS, Promega Corporation, Mannheim, Germany) was used to determine viable cells in proliferation at one hour after thawing and at day one, two, and three. Figure 5 illustrates the absorption of viable cells in proliferation after vitrification. DMEM = DMEM Ham’s F-12 Gln+, 20% FBS, and 3M of one of the CPAs; K^+^TiP = K^+^TiP. 0.5M sucrose, 20% FBS, and 3M of one of the CPAs; DMSO = dimethyl sulfoxide; EG = ethylene glycol; PG = propylene glycol; GLY = glycerin; * = significant (p < 0.05); ** = medium significant (p < 0.01); au = absorbance units.

Three days after thawing, the highest absorbance of cells frozen by utilizing DMEM Ham’s F-12 Gln+ as the cryopreservation medium was 2.93 ±0.25 x10^-7^au (277.6 ±23.2% relative to unfrozen control at day 0) with DMSO, and then 0.98 ±0.46 x10^-7^au (92.7 ±44%) with GLY, 0.27 ±0.11 x10^-7^au (26.1 ±10%) with EG, and 0.12 ±0.01 x10^-7^au (11.4 ±1.3%) with PG as the CPA. The highest absorbance after the thawing of cells frozen by utilizing K^+^TiP as the cryopreservation medium was 3.25 ±0.36 x10^-7^au (308.2 ±34.4%) with GLY, and then 2.65 ±0.16 x10^-7^au (250.6 ±14.9%) with DMSO, 0.28 ±0.09 x10^-7^au (26.6 ±8.1%) with PG, and 0.15 ±0.07 x10^-7^au (14 ±7%) with EG as the CPA. Fig 5 illustrates these findings.

**Fig 6:**
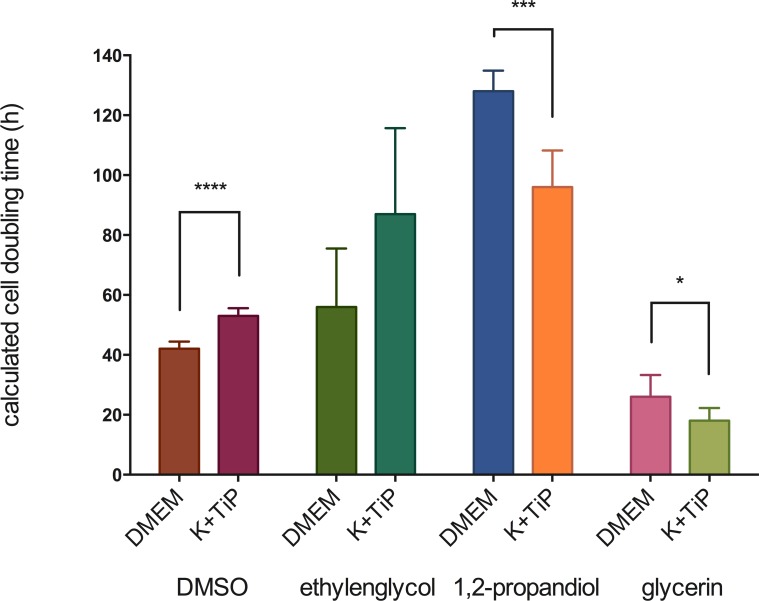
Cell doubling-time after vitrification. Figure 6 illustrates MHEC-5T doubling-time after vitrification. DMEM = DMEM Ham’s F-12 Gln+, 20% FBS, and 3M of one of the CPAs; K^+^TiP = K^+^TiP. 0.5M sucrose, 20% FBS, and 3M of one of the CPAs; * = significant (p < 0.05); *** = highly significant (p < 0.001); **** = highest siginificance (p < 0.0001).

The fastest MHEC-5T doubling-time of cells previously frozen by utilizing DMEM Ham’s F-12 Gln+ as the cryopreservation medium was 26 ±7.3h with GLY, and then 42 ±2.4h with DMSO, 56 ±19.5h with EG, and 128 ±6.9h with PG as the CPA. The doubling-time of cells frozen by utilizing K^+^TiP as the cryopreservation medium was 18 ±4.2h with GLY, 53 ±2.6h with DMSO, 87 ±28.7h with EG, and 96 ±12.2h with PG as the CPA. Fig 6 illustrates these findings.

**Fig 7:**
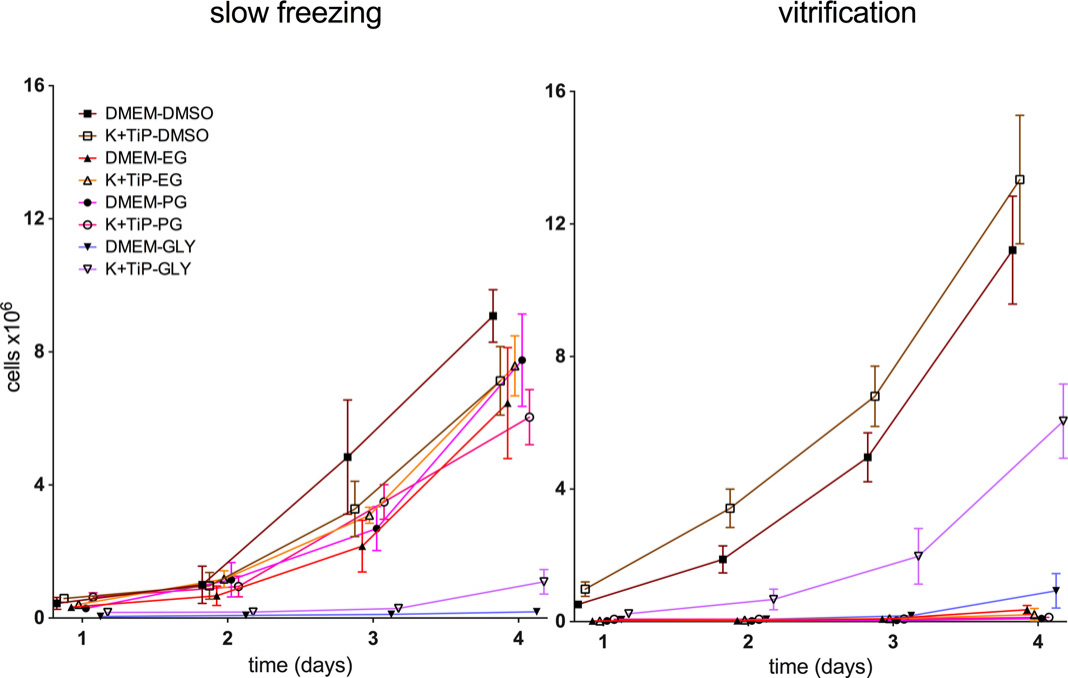
Culture of thawed cells after slow freezing and vitrification. Cells in culture after thawing were counted in phase contrast photographs with adobe illustrator CS5 (Adobe Systems, Delaware, USA). DMEM = DMEM Ham’s F-12 Gln+, 20% FBS, and 1.5M (slow freezing) / 3M (vitrification) of one of the CPAs; K^+^TiP = K^+^TiP, 0.5M sucrose, 20% FBS, and 1.5M (slow freezing) / 3M (vitrification) of one of the CPAs. DMSO = dimethyl sulfoxide; EG = ethylene glycol; PG = propylene glycol; GLY = glycerin.

All frozen cells could be cultured until confluence in the 25 cm^2^ cell culture flasks after thawing. Confluence was reached as early as day 3 by cells previously frozen by utilizing K^+^TiP as the cryopreservation medium and DMSO as the CPA. On day 4, confluence was reached by cells previously frozen by utilizing DMEM Ham’s F-12 Gln+ as the cryopreservation medium and DMSO as the CPA and by utilizing K^+^TiP as the cryopreservation medium and GLY as the CPA. On day 6, confluence was reached by cells previously frozen by utilizing DMEM Ham’s F-12 Gln+ as the cryopreservation medium and GLY as the CPA. On day 8, confluence was reached by cells previously frozen by utilizing K^+^TiP as the cryopreservation medium and EG as the CPA. On day 9, confluence was reached by cells previously frozen by utilizing DMEM Ham’s F-12 Gln+ as the cryopreservation medium and EG as the CPA. On day 10, confluence was reached by cells previously frozen by utilizing K^+^TiP as the cryopreservation medium and PG as the CPA. Only on day 18 was confluence reached by cells previously frozen by utilizing DMEM Ham’s F-12 Gln+ as the cryopreservation medium and PG as the CPA. Fig 7 illustrates these findings.

## Discussion

Our aim was to compare the promising but sparsely examined technique of vitrification with the standard slow freezing technique. To optimize the media used in vitrification, we introduced a modified organ preservation solution that showed excellent results in the prevention of iron-dependent cold-induced injury during the cold storage of vessels. K^+^TiP contains 131 mM potassium in order to terminate the membrane resting potential during freezing. With a terminated resting potential, the O_2_ consumption of the cells decreases [18]. Wille et al. have reported significantly lower damage to endothelial cells by using potassium as the main cation, instead of sodium [19]. High extracellular potassium is responsible for the prevention of the K^+^-outflow of the cell [20, 21], which leads to its apoptosis [22]. The pH of K^+^TiP was adjusted to 7.0, as a mild acidosis is protective against multiple mechanisms of cellular damage [23].

TiProtec (Dr. Franz Köhler Chemie GmbH, Bensheim, Germany) organ storage solution contains several ingredients that counteract the damage mechanism of cold storage. TiProtec was used as the basis for K^+^TiP. It contains the following additions:

Several studies have shown that the cold-induced injury of several cultured cell types is mediated via the iron-dependent formation of reactive oxygen species (ROS). Chelatable ‘‘redox-active” iron plays the major role in the development of cold-induced apoptosis [24, 25]. To prevent cold-induced injury, the hydroxamic acid derivative LK 614 is therefore included in TiProtec. The aromatic ring system gives the molecule a sufficiently high lipophilicity to permeate membranes. Glucose is added as a substrate for the glycolytically highly active endothelial cells [26]. To prevent the formation of non-specific pores during hypoxia followed by sodium influx, TiProtec contains the amino acid glycine and the structurally related alanine [27, 28]. Aspartate and α-ketoglutarate are included as intermediates of the citric acid cycle or their precursors and tryptophan is present in TiProtec [28].

Sucrose at 0.5 M sucrose has been added to VS because its addition reduces the concentration of permeable CPAs needed to achieve the glassy state and to avoid devitrification [13]. In addition, by dehydrating the cells, sucrose helps to avoid excessive swelling and osmotic shock during the removal of permeating cryoprotectants. Macromolecular CPAs also have a specific role in the glass-forming trend. They tend to have increasingly high viscosities at low temperature, and so the solution containing macromolecules effectively becomes too viscous to allow water molecules to join growing ice crystals [29].

DMSO is used routinely for cryopreservation and in vitrification. Because of the relatively good results, other CPAs succh as EG, PG, or GLY, which are considerably less cell toxic, are rarely used. The cell toxicity of the four tested CPAs can be ranked as follows: DMSO *>* EG ≈ PG *>* GLY [5]. In our opinion, the less toxic CPAs have the important benefit that cells are considerably less vulnerable with regard to the time necessary for the freezing process. Different workers need different time spans in order to freeze their cells and, therefore, the influence of the toxic CPAs used during this freezing process might underlie extensive differences in the cells obtained on thawing.

The cryopreservation process involving vitrification is fast and reliable. The precooled DMEM Ham’s F-12 Gln+ is added to the centrifuged cells in a cryo vial, followed by the prepared and precooled VS. The mixture is pipetted up and down two to three times to ensure good mixing, and then the cryo vial is tightly capped and directly plunged into LN_2_. This process can be conducted within less than 15 seconds per vial and can be performed by various researchers within the same time span. For slow freezing, the mixing process is the same, but the first prepared vial needs to rest in the CoolCell or on ice until the last one is ready, and everything can be stored in -20°C.

Cell survival is normally evaluated by using live/dead staining with membrane-permeant molecules that are cleaved by esterases in live cells to yield cytoplasmic fluorescence and the membrane-impermeant dye, propidium iodide. Because propidium iodide is cell-toxic, this method is highly time-dependent, not very sensitive, and difficult to reproduce. Therefore, we developed and used a new time-independent and cell-count-adjusted method to evaluate cell survival and the number of viable cells in proliferation after thawing.

Because of the large variability of the amount of cells that can be plated into a particular well or cryo vial, we decided first to minimize this error by repetitive gentle vortex of the cell suspension prior to the plating of each well; to this end, we used a multistep pipette (Eppendorf repeater stream combitip advanced, Hamburg, Germany). Our results indicated, that in spite of great care being taken, with repetitive gentle vortexing and the use of a multistep pipette, the number of cells per cryo vial varied between 279,000 and 590,000 (median 461,000, standard deviation 67,000, n = 32). To detect the exact cell number per cryo vial, the DNA content was measured according to Kim et al. by using Hoechst 33258 dye. This assay is stable for at least 2 h and sensitive to as little as 6 ng of DNA or equivalently less than 1,000 cells [17]. We finally evaluated not the simple results achieved from CellTiter 96® A_Queous_ One Solution Cell Proliferation Assay, but those normalized by building a quotient of fluorescence intensity and cell number per cryo vial. The result is a ratio of measured absorbance that is directly proportional to the number of living cells in culture compared with the total cell number.

In slow freezing, we used a CoolCell, because it precisely controls air flow and heat removal to ensure standardized cell freezing runs and no variability in the cryopreservation period. The elimination of alcohol from the cryopreservation process means no maintenance, no ongoing expense, no waste, and no variability [30].

After use of the standardized controlled-rate -1°C/minute protocol, the cell recovery rates after thawing could be ranked in the following order with respect to CPA GLY < EG ≈ PG < DMSO. The use of DMEM Ham’s F-12 Gln+ as the cryopreservation medium showed a statistical benefit compared with K^+^TiP with DMSO or PG as the CPA. Vitrification gave the following order with regard to cell recovery rates after thawing: PG < EG << GLY ≈ DMSO. In this case, the use of K+TiP as the cryopreservation medium showed a statistical benefit compared with DMEM Ham’s F-12 Gln+ with DMSO and particularly PG and GLY as CPA. Finally, the best cryopreservation method was vitrification with VS containing K^+^TiP, 40% FBS, 0.5 M sucrose, and 6M CPA. With regard to cell recovery immediately after thawing, DMSO was the most suitable CPA (99 ±0.5%). The most viable cells in proliferation after three days of culture could be achieved by using GLY (308 ±34%). However, against expectation, K^+^TiP had no benefit in the slow freezing methods. We conclude that, only in vitrification, is the termination of membrane resting potential beneficial. In slow cooling, the termination of the membrane resting potential is harmful to cells, and therefore, GLY as a low-toxic CPA, shows good long-term results when used in slow freezing technology with K^+^TiP as the cryopreservation medium. The preservation of surface markers is possibly better with low-toxic CPAs. To clarify this, further studies with fluorescence-activated cell sorting analysis are necessary. Additional investigation is also needed to determine whether this effect is influenced by the potassium concentration or another modification made compared with DMEM Ham’s F-12 Gln+. The various methods of assessment of recovery and the various cryopreservation media used by other groups make direct quantitative comparisons of the various results difficult or even impossible.

## Supporting Information

S1 Data Set Minimal data set. The minimal data set includes the following files: results_freezing.xls, results_vitrific.xls and results_cells_25cm2_counted.xls.
(ZIP)
